# The Role of C-Terminal Crosslinking Telopeptide of Type II Collagen (CTX-II) After Anterior Cruciate Ligament Reconstruction: A Systematic Review

**DOI:** 10.7759/cureus.103044

**Published:** 2026-02-05

**Authors:** Ali Naderi, S. Ali Ghasemi, Andrea Fabregas, Gene Shaffer, James Raphael

**Affiliations:** 1 Department of Orthopaedic Surgery, Philadelphia College of Osteopathic Medicine, Philadelphia, USA; 2 Department of Orthopaedic Surgery, Einstein Healthcare Network, Philadelphia, USA; 3 Department of Orthopaedics, Universidad Central del Caribe, Bayamón, USA

**Keywords:** acl reconstruction, cartilage degradation, ctx-ii, osteoarthritis, urinary biomarker

## Abstract

Anterior cruciate ligament (ACL) injuries are known to accelerate cartilage degradation and predispose patients to early-onset osteoarthritis. C-terminal cross-linked telopeptide of type II collagen (CTX-II), a biomarker of cartilage breakdown, can be detected in various body fluids, including urine, offering a non-invasive method for evaluating cartilage degeneration. This systematic review aimed to assess existing evidence on urinary CTX-II (uCTX-II) levels following ACL reconstruction (ACLR). A systematic search of two medical databases was conducted in accordance with PRISMA guidelines. Studies were screened for inclusion based on their assessment of CTX-II in human subjects undergoing ACLR, and methodological quality was evaluated using the Modified Coleman Methodology Score (MCMS). Four studies met the inclusion criteria, with methodological quality ranging from fair to good. However, the limited number of studies and variability in time points prevented consistent analysis of uCTX-II trends postoperatively. While current evidence is insufficient to establish temporal trends or reference ranges, uCTX-II remains a promising non-invasive biomarker for monitoring cartilage degradation in ACLR patients. These findings should be interpreted cautiously due to the limited number of studies, heterogeneity in study design and postoperative time points, and the restricted database search. Further longitudinal studies are needed to validate its clinical utility.

## Introduction and background

Anterior cruciate ligament (ACL) injuries are very common orthopaedic injuries that can occur across different age groups, with a typical mechanism of injury consistent with an abrupt change of direction or a stopping motion of the knee relative to a planted foot. ACL tears can also occur after direct contact, usually with a direct blow to the lateral aspect of the leg. According to the American Academy of Orthopedic Surgeons, there are around 200,000 ACL injuries per year in the United States [1,2].

Regardless of how an ACL tear is treated, there are almost always long-term consequences. One commonly studied and important long-term consequence is early-onset cartilage degradation due to instability of the knee, which in turn leads to altered loading of the joint. This ultimately leads to early-onset osteoarthritis (OA). During this entire process, there are robust inflammatory processes that contribute to the aforementioned cartilage damage [1,3]. More specifically, when the ACL is injured, the inflammatory response leads to various enzymes being released that will break down existing cartilage. After acute ACL injury, there are marked elevations in inflammatory cytokines such as interleukin-1 and other catabolic enzymes such as matrix metalloproteinases (MMPs). In addition to these inflammatory cytokines, there are additional markers of cartilage damage and degradation [1,4,5].

Included in the many cartilage degradation byproducts, the C-terminal crosslinking telopeptide of type II collagen (CTX-II) is a biomarker that has been used to monitor OA [1,5]. CTX-II is a breakdown product of type II collagen, which is the primary collagen in articular cartilage. This byproduct is produced when the aforementioned catabolic enzymes act on the articular cartilage. 

CTX-II has been studied in serum, synovial fluid, and urine as a biomarker of osteoarthritis progression, with reported associations to disease severity [6]. While serum and synovial fluid measurements can be invasive and impractical for longitudinal monitoring, urine sampling offers a more feasible non-invasive alternative following ACL reconstruction. 

However, elevations in urinary CTX-II may reflect multiple biological processes beyond cartilage degeneration alone. Factors such as exercise-induced collagen turnover, postoperative inflammation, and acute tissue remodelling following ACL reconstruction may influence uCTX-II levels independent of progressive osteoarthritic change. Distinguishing these transient or non-degenerative influences from true cartilage breakdown remains a key unresolved challenge and represents an important gap in the current literature.

Given the association between ACL injury and cartilage degradation, this review systematically evaluates the current literature on urinary CTX-II (uCTX-II) as a marker of cartilage breakdown following ACL reconstruction. The objective of this review is to evaluate the current evidence of CTX-II in the context of ACL Reconstruction, identify gaps in the existing literature, and provide guidance for future research efforts. 

Biomarkers are measurable biological molecules that reflect ongoing tissue processes, such as cartilage degradation, and may provide clinicians with a non-invasive method to monitor joint health over time. Although several biomarkers have been proposed to assess cartilage breakdown, their clinical utility remains limited due to inconsistent findings and the absence of standardized postoperative trends. In particular, while the C-terminal cross-linked telopeptide of type II collagen (CTX-II) has been studied in osteoarthritis and acute knee injury, its longitudinal behavior following anterior cruciate ligament reconstruction has not been clearly defined. This gap limits the translation of urinary CTX-II into routine clinical decision-making and postoperative monitoring. Therefore, the purpose of this systematic review is to evaluate the current evidence on urinary CTX-II levels following ACL reconstruction and to identify gaps that should be addressed in future longitudinal studies.

## Review

Methods

Search Strategy and Selection Criteria 

**Figure 1:**
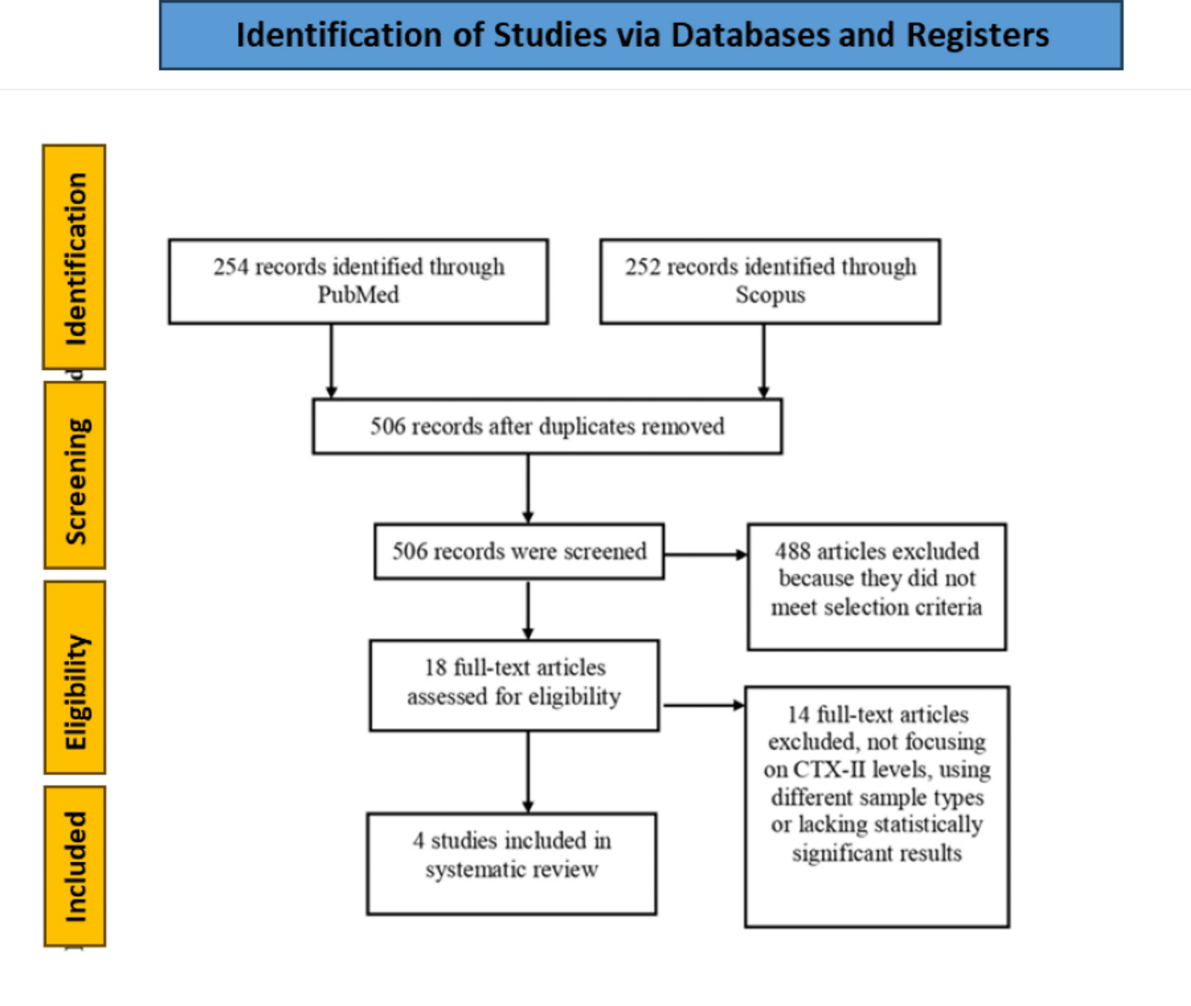
PRISMA (Preferred Reporting Items for Systematic Reviews and Meta-Analyses) flow diagram.

A systematic review of the available literature was performed using Preferred Reporting Items for Systematic reviews and Meta-Analyses (PRISMA) [7]. A detailed PRISMA flow chart is provided in Figure 1. Two independent reviewers completed the search individually. The search was completed on September 3, 2024, using two databases: PubMed and Scopus. Search terms included “knee” AND “anterior cruciate ligament” AND “biomarker”. We included studies that used human subjects as well as studies that were written in English. An initial search of the two databases yielded 506 articles. After removing duplicates (none were found), we reviewed 506 articles and determined that 18 met the initial eligibility criteria. 

Inclusion criteria were as follows: original research articles involving human subjects; studies assessing anterior cruciate ligament (ACL) injury or ACL reconstruction; studies reporting C-terminal crosslinking telopeptide of type II collagen (CTX-II) levels; and articles written in English.

Exclusion criteria included: studies focusing exclusively on biomarkers unrelated to cartilage degradation or without CTX-II measurement; articles not reporting outcomes relevant to ACL injury, reconstruction, or subsequent cartilage degradation; and studies with insufficient follow-up or poor methodological quality preventing meaningful interpretation. Information regarding medication use, including non-steroidal anti-inflammatory drugs, corticosteroids, or exogenous collagen supplementation, was inconsistently reported across studies and therefore could not be accounted for as an exclusion criterion or controlled variable.

Although only two databases were searched, this strategy was intentionally designed to identify studies specifically reporting C-terminal cross-linked telopeptide of type II collagen (CTX-II) measurements in human subjects following anterior cruciate ligament injury or reconstruction. Studies evaluating non-urinary biomarkers or mechanistic inflammatory pathways were reviewed for contextual purposes but were not eligible for inclusion in the primary quantitative analysis.

However, upon further evaluation, we applied stricter inclusion criteria that considered factors such as the study design quality, the specificity of the biomarkers studied, and the relevance of the outcomes to our research question. Additionally, some studies were excluded due to insufficient data, lack of rigorous analysis, or having no follow-up recorded. As a result, only four of the 18 eligible studies were ultimately included in our final analysis.

Due to heterogeneity in study design, biomarker reporting, and postoperative time points, a quantitative synthesis or meta-analysis was not performed, and findings were synthesized descriptively.

The methodological quality of the studies was assessed using the Modern Coleman Methodology Scores (MCMS) [8], and the risk of bias for each included study was evaluated using the risk of bias in non-randomized studies of interventions (ROBINS-I) tool [9]. MCMS tool consists of fifteen items and provides a scaled score ranging from 0 to 100, where 85-100 indicates excellent quality, 70-84 represents good quality, 55-69 indicates fair quality, and scores below 55 are considered poor. The MCMS ratings for the four included studies are presented in Table 1. 

**Table 1:** Methodological quality assessment of included studies using the Modified Coleman Methodology Score (MCMS).

Author (year)	Sample size	MCMS score	Quality rating
Hagemans [10]	152	65	Fair
Chmielewski [11]	56	62	Fair
Tourville [12]	67	68	Fair
Chmielewski [13]	24	72	Good

Risk of Bias Assessment

The risk of bias for each included study was evaluated using the risk of bias in non-randomized studies of interventions (ROBINS-I) tool [9]. The risk of bias was performed and assessed across seven domains: bias due to confounding, bias in the selection of participants, bias in the classification of interventions, bias due to deviations from intended interventions, bias due to missing data, bias in the measurement of outcomes, and bias in the selection of the reported result [9]. Each domain was rated as low, moderate, serious or critical risk, and overall study assessment is summarized in Table 2.

**Table 2:** Risk of bias assessment of included studies using the ROBINS-I tool.

Study	Bias due to confounding	Bias in the selection of participants	Bias in the classification of interventions	Bias due to deviations from intended interventions	Bias due to missing data	Bias in the measurement of outcomes	Bias in the selection of the reported result	Overall risk
Hagemans[10]	Moderate	Low	Low	Low	Moderate	Low	Moderate	Moderate
Chmielewski [11]	Moderate	Low	Low	Moderate	Low	Low	Moderate	Moderate
Tourville [12]	Moderate	Low	Low	Low	Low	Low	Moderate	Moderate
Chmielewski [13]	Low	Low	Low	Moderate	Low	Low	Moderate	Low to moderate

Results 

Based on our search strategy and selection criteria, we were left with 18 publications. These articles ranged from 2003 to as recent as 2024. Many identified studies evaluated multiple biomarkers, with CTX-II reported as a secondary outcome rather than the primary focus. 

Given the specific focus on urinary CTX-II trends following ACL reconstruction, many of these studies did not meet the inclusion criteria for analysis. A handful of the studies looked at other samples, such as synovial fluid or serum, while others did not provide specific time points and values, but instead just provided trends. Furthermore, some of the studies did not produce statistically significant results. 

Of the 18 publications in our search, only four were eligible for inclusion in our analysis. Three of the studies achieved a fair MCMS score, and one achieved a good score. This indicated that the overall quality of the study was fair to good. Due to substantial heterogeneity in study design, outcome metrics, clinical context, and timing of urinary CTX-II measurement, results were synthesized using a structured descriptive narrative approach rather than subgroup analysis or direct comparison.

Studies assessing CTX-II exclusively in serum or synovial fluid, reporting qualitative trends without quantitative urinary values, or lacking clearly defined postoperative time points were excluded from the primary analysis but are referenced selectively to provide biological and clinical context. Across the four studies included in our analysis, there were a total of 299 subjects. Hagemans et al. (2021) studied a total of 152 subjects with ACL tears who had a median age of 25 (IQR 21-32) [10]. 65.8% of these total subjects were males, and they obtained 62 urine reference samples with a median age of 25 (IQR 22-36). Of the total 152 subjects, these were further divided into nonoperative and operative subgroups. 98 subjects received surgery, and of these subjects, 68.4% were male (67/98) with a median age of 23 (IQR 20-28). In the surgery subgroup, the median time from injury to surgery was 24 weeks (IQR 14-36 weeks). Time points when data was acquired include baseline and roughly 1 and 2 years after injury (56 and 108 weeks). In the surgery subgroup, they reported median urine CTX-II values of 906 ng/mmol creatinine (IQR: 446-1821, N=96), 740 ng/mmol creatinine (IQR: 410-1456, N=92), and 655 ng/mmol creatinine (IQR: 382-1002, N=87) at baseline, one, and two years, respectively. In the nonoperative group, they reported median urine CTX-II levels of 417 ng/mmol creatinine (IQR: 249-1083, N=54), 365 ng/mmol creatinine (IQR: 133-649, N=48), and 395 ng/mmol creatinine (IQR: 151-584, N=49) at baseline, one, and two years, respectively. 

Chmielewski et al. (2012) studied a total of 56 subjects, 28 of whom underwent ACLR, and the other 28 were age and sex matched controls [11]. Both subgroups included 50% male subjects (28/56 males total), and the ACLR subgroup had a mean age of 19.6 (SD 4.5), while the control group had a mean age of 19.9 (SD 4.3). Average time from injury to surgery was a mean of 69.6 days (SD 64.3), with data being collected at four different time points: 4, 8, 12, and 16 weeks post-ACLR. The ACLR subgroup reported mean urinary CTX-II levels of 3521.2 ng/mmol (SD 4217.5), 3332.8 ng/mmol (SD 3795.1), 3190.5 ng/mmol (SD 3317.9), and 2850.7 ng/mmol (SD 3185.5) at 4, 8, 12, and 16 weeks, respectively. The control group reported a mean uCTX-II level of 2827.8 ng/mmol (SD 5033.7) across all time points. 

Tourville et al. (2013) studied a total of 67 subjects [12]. 35 subjects were in an ACLR group with a mean age of 28.8, and the other 32 subjects made up the control group with a mean age of 26.8. Timing of ACLR occurred at a mean of 70.1 days after injury (Range 18-155) with data points that were measured at baseline, one and four years. The ACL group was also subdivided into Normal and Abnormal Joint Space Width (JSW) groups. Of note, data was reported as a ratio of urinary CTX-II to serum CPII (uCTX-II/sCPII). In the normal JSW group, they reported a mean uCTX-II/sCPII ratio of 0.5 (SD 0.3) and 0.4 (SD 0.6) at 1 and 4 years, respectively. They also reported median uCTX-II/sCPII values of 0.3 (Range 0.1-1.0) and 0.2 (Range 0.1-2.3) at 1 and 4 years, respectively. 

In the abnormal JSW group, they reported mean uCTX-II/sCPII ratios of 0.7 (SD 0.4) and 0.5 (SD 0.6) at 1 and 4 years, respectively. They also reported median uCTX-II/sCPII values of 0.8 (Range 0.2-1.2) and 0.4 (Range 0.1-2.2) at 1 and 4 years, respectively. The control group reported a mean uCTX-II/sCPII ratio of 0.3 (SD 0.4) and a median uCTX-II/sCPII ratio of 0.2 (Range 0.03-2.2). 

Chmielewski et al. (2016) evaluated urinary CTX-II levels in 24 patients following ACL reconstruction within the context of a low- versus high-intensity exercise rehabilitation program over eight weeks [13]. This study included 62.5% male subjects (15/24), and the mean age for the low and high intensity groups was 20.7 (SD 4.9) and 19.3 (SD 3.8), respectively. The mean time from injury to ACLR for the low and high intensity groups was 5.8 (SD 3.9, Range (2-14)) and 11.7 (SD 9.5, Range (3-36)) weeks, respectively. Chmielewski et al. (2016) reported uCTX-II levels before and after intervention [11]. In the low intensity group, they reported a mean uCTX-II (log) of 3.34 (SD 0.47) ng/mmol and 3.29 (SD 0.54) ng/mmol before and after intervention, respectively. In the high intensity group, they reported a mean uCTX-II (log) of 3.45 (SD .048) ng/mmol and 3.36 (SD 0.46) ng/mmol before and after intervention, respectively. Of importance, they noted a p-value of 0.856 for this set of data. 

Discussion

Upon further review of the current data in the literature, it is evident that there is limited information regarding urinary CTX-II values following ACLR. Importantly, the biological interpretation of urinary CTX-II varies depending on the postoperative stage at which it is measured. In the early postoperative period, elevations in uCTX-II may reflect acute cartilage injury, surgical inflammation, or altered joint loading during rehabilitation, whereas measurements obtained years after reconstruction may more closely represent longer-term cartilage degeneration and osteoarthritic remodelling.

This discussion first summarizes findings from studies directly reporting urinary CTX-II measurements following ACL reconstruction, followed by contextual literature addressing inflammatory mechanisms and non-urinary biomarkers relevant to cartilage degradation. Mouton et al. (2024) recently conducted a systematic review that evaluated a variety of biomarkers for early knee OA diagnosis [14]. While they identified several potential candidates, including CTX-II. Although the study identified several potential candidates, it concluded that no biomarker is currently reliable enough to differentiate early OA from healthy controls. Among the four studies that provide urinary CTX-II values after ACL reconstruction, there are a few pitfalls that make analysis of the data difficult. One barrier is the wide variety of time points used in each study; Chmielewski et al. (2012) reported data points at 4, 8, 12, and 16 weeks after ACLR, while Tourville et al. (2013) reported data points at 1 and 4 years [11,12]. Hagemans et al. (2021) reported their initial time point at 3-25 weeks after injury, then 56 and 108 weeks [10]. Furthermore, Chmielewski et al. (2016) reported data points before and after ACLR [13]. With the wide range of time points, it is clearly difficult to analyze the data and compare the urinary CTX-II. Having no overlap in time points, it is virtually impossible to trend or create a predictable course of the data. 

Biologically, divergent urinary CTX-II findings following ACL reconstruction likely reflect a combination of time-dependent cartilage remodelling, postoperative inflammation, and mechanical loading during rehabilitation rather than isolated cartilage degeneration. Early elevations in uCTX-II may represent acute collagen turnover related to surgery and rehabilitation intensity, whereas later measurements may better reflect chronic cartilage breakdown processes similar to those described in osteoarthritis. These dynamics are consistent with broader osteoarthritis biomarker literature, in which CTX-II levels vary according to disease stage, mechanical stress, and inflammatory activity rather than reflecting a single pathologic process.

Another issue that is raised with the data is that the urinary CTX-II values (uCTX-II) reported are not easily standardized based on the relative volume inside the knee joint. Meaning some patients could have higher values; however, this could reflect a higher or lower overall volume in their respective knee joint. These values could essentially vary from patient to patient due to a dilution effect. Two papers, Tourville et al. (2013) and Cattano et al. (2017), reported CTX-II levels as a ratio of CTX-II/CPII [12,15]. Reporting this data as a ratio could be a great way to eliminate the confounding variable of relative knee volume and create a more standardized value for true CTX-II levels [15-17].

Studies not directly reporting urinary CTX-II following ACL reconstruction were included selectively to provide biological and mechanistic context rather than to inform the primary analysis. Several studies have evaluated CTX-II in serum or synovial fluid rather than urine. Although these studies do not directly report urinary CTX-II measurements following ACL reconstruction, they provide important mechanistic insight into cartilage degradation and the inflammatory environment after ACL injury. These studies include Izaguirre et al., Sullivan et al., Lohmander et al., Hunt et al., Cattano et al., Catterall et al., and Amano et al. [15,18-23]. Other studies briefly referenced CTX-II without reporting quantitative values, including King et al., Zou et al., and Struglics et al. [24-26]. While CTX-II was not the primary outcome in these studies, their findings help contextualize the biological pathways underlying cartilage breakdown.

Regardless, in our entire search, only four papers fit our ultimate criteria of providing urinary CTX-II levels at a measurable time point after ACLR (Table 3). It is evident that there is not much data on CTX-II in this subset of the population, and there is room for further research. The ultimate goal is to identify a trend for uCTX-II that can be followed longitudinally. At this point, we have deemed that there is not enough data to adequately analyze the data in this systematic review. The limited number of studies and heterogeneity in study design, biomarker reporting, and postoperative time points underscore the narrow scope of the current evidence base and highlight the need for standardized, longitudinal investigations focused specifically on urinary CTX-II following ACL reconstruction. Further research is needed to provide sufficient data at time points, which can then be extrapolated and analyzed. 

**Table 3:** The table presents the four papers included in our analysis that discussed uCTXII and ACL reconstruction.

Author	Year	Study design	Sample type	Sample size	Age (mean)	Gender
Chmielewski [11]	2012	Longitudinal	Urine	56	19.6	28 males
Chmielewski [13]	2016	Randomized control trial	Urine	24	20	15 males
Hagemans [10]	2021	Descriptive	Urine	152	25	100 males
Tourville [12]	2013	Longitudinal	Urine	67	28.8	Not reported

## Conclusions

ACL injuries can play a major role in early cartilage damage and the development of osteoarthritis. Tracking cartilage degradation may help improve understanding of disease progression, and this can be assessed using cartilage degradation biomarkers such as C-terminal crosslinking telopeptide of type II collagen (CTX-II). Urinary CTX-II offers a minimally invasive approach for monitoring cartilage turnover following ACL reconstruction. However, the current evidence base is limited. Given the small number of heterogeneous studies and absence of quantitative synthesis, findings should be interpreted as hypothesis-generating rather than practice-changing. Based on the available evidence, urinary CTX-II may be most informative when measured longitudinally, with early postoperative measurements reflecting acute and rehabilitation-related cartilage turnover and later measurements potentially providing insight into longer-term degenerative risk. At present, available evidence does not support the routine clinical application of urinary CTX-II following ACL reconstruction, and its role should be considered investigational rather than clinically actionable. Further longitudinal studies are needed to establish baseline trends and reference ranges for urinary CTX-II after ACL reconstruction.
